# Toward a Molecular Classification of Colorectal Cancer: The Role of Microsatellite Instability Status

**DOI:** 10.3389/fonc.2013.00272

**Published:** 2013-10-31

**Authors:** Karl Heinimann

**Affiliations:** ^1^Department of Biomedicine, Research Group Human Genomics, Basel, Switzerland; ^2^Medical Genetics, University Hospital Basel, Basel, Switzerland

**Keywords:** microsatellite instability, mismatch repair, Lynch syndrome, hereditary non-polyposis colon cancer, colorectal cancer, therapy, prognosis

## Abstract

Microsatellite instability (MSI) is the molecular hallmark of DNA mismatch repair deficiency. Since its initial description in colorectal cancer (CRC) in 1993 and its association with Lynch syndrome, the most common inherited cancer predisposition world-wide, accumulating evidence suggests that MSI status may also be of concrete prognostic and predictive value in the management of sporadic CRC. This mini review aims at providing a concise survey of the molecular basis and the multifaceted role(s) of MSI status in today’s clinical practice.

## Introduction

Twenty years ago, in 1993, in search for molecular clues to the pathogenesis of colorectal cancer (CRC) several research groups had made an intriguing observation: widespread somatic alterations at short, repetitive DNA sequences, referred to as replication error (RER+) phenotype or, more specifically, microsatellite instability (MSI) (1–3). They detected this novel form of genomic instability in about 10–15% of sporadic, predominantly proximally located, colorectal carcinomas as well as in most (>90%) of those from patients with the most common inherited cancer predisposition, Lynch syndrome (LS), also referred to as Hereditary Non-Polyposis Colon Cancer (HNPCC). The discovery allowed to link the hereditary disorder to a defect in a key DNA metabolic pathway, the mismatch repair (MMR) system, which had previously been known to cause MSI in *Saccharomyces cerevisiae*, and eventually opened up a new field in cancer research (4).

## The Molecular Basis of MSI

Microsatellites, also referred to as short tandem repeats (STRs), consist of a few to several thousands of tandemly repeated motifs made up of one (mono) up to six (hexa) nucleotides and are thought to account for approximately 3% of the human genome (5). Given their number and the fact that they are scattered throughout the genome, their (hyper)variability in length coupled with a high degree of heterozygosity made them ideal polymorphic markers for genome mapping, population genetics, and genetic linkage studies as well as indispensable tools in forensics and transplantation medicine (“DNA fingerprint”).

Due to their repetitive sequence structure, it is not surprising that microsatellites exhibit a particularly high mutation rate compared to non-repetitive, unique DNA stretches. During replication of the nuclear genome DNA polymerases, due to slippage, often fail to correctly duplicate such microsatellite tracts. The resulting insertion/deletion loops can consequently lead to insertions or deletions of repeats, thereby altering the length of the respective microsatellite (replication error, RER). In eukaryotes these errors are corrected by the DNA MMR system: its heterodimers MSH2/MSH6 and MSH2/MSH3 detect the replication error (licensing step) and recruit the MLH1/PMS2 complex through which degradation of the mutated stretch and resynthesis are initiated (4). As originally observed in yeast, defects in MMR consequently result in the genome-wide accumulation of mutations at microsatellite loci, MSI, which has also been referred to as a “mutator phenotype” (6).

## Assessment of MSI

Since MSI analysis of colorectal tumors provided a straight-forward, though indirect, means to identify LS patients great attention was given to the development of selection criteria and microsatellite markers to be used for testing. In 1996 and 2002 the National Cancer Institute in Bethesda, MD, held workshops in which the Bethesda guidelines for the identification of LS patients were defined (7, 8). MSI testing is recommended in patients who meet one of the following criteria: (a) diagnosed with CRC before age 50 years, (b) synchronous or metachronous CRC or other LS-related tumors, (c) CRC with typical MSI-high morphology and diagnosed before age 60 years, (d) CRC in one or more first-degree relatives with CRC or other LS-related tumors, one being diagnosed before age 50 years, and (e) CRC with two or more relatives with CRC or other LS-related tumors, regardless of age. The revised Bethesda guidelines thus incorporate personal as well as family history and pay attention to the fact that LS actually comprises a spectrum of different tumor types (endometrial, gastric, etc.).

To assess the presence of MSI in a given tumor the NCI workshop recommended to analyze a panel of five microsatellites including two mono- (BAT25, BAT26) and three di-nucleotide markers (D2S123, D5S346, and D17S250); depending on the mutational pattern, a secondary panel including additional mononucleotide (e.g., BAT40) and/or complex microsatellites (e.g., MYCL) should be tested (8). Alternatively, a panel of five quasimonomorphic mononucleotide repeats can be used which display even better sensitivity and which may obviate the need for normal tissue for comparison (9). Depending on the number of microsatellite markers displaying novel alleles, MSI can subsequently be rated as MSI-high (MSI-H, >2 out of 5 markers), MSI-low (MSI-L, 1 out of 5), or microsatellite stable (MSS, 0 out of 5).

The molecular dissection of CRCs into MSI-H and MSS tumors allowed to delineate two major, virtually exclusive pathways of genetic instability: chromosomal instability, which results in aneuploidy and is present in about 85% of CRC, and MSI. Whether MSI-L CRC constitute a pathogenetic class of their own is still a matter of debate: with regard to clinical, biological, and morphological parameters they closely resemble those of MSS CRC. Since the analytical sensitivity to detect MSI-L is dependent on the number of microsatellite markers analyzed, result interpretation, and comparison between different studies investigating MSI-L and MSS tumors are heavily compromised; furthermore, extensive genotyping efforts have failed to demonstrate fundamental differences (10, 11). Given these unresolved issues, a molecular subdivision into MSI-L and MSS CRC does currently not seem appropriate.

## Role of MSI in Lynch Syndrome

The discovery of MSI in the majority of LS-related CRC led by analogy to a similar biochemical defect in yeast to the identification of the underlying MMR germ line mutations in MLH1, MSH2, MSH6, and PMS2. Heterozygous carriers of a MMR gene alteration are at a greatly increased lifetime risk to develop cancers of the LS tumor spectrum, mainly CRC (25–70%) and endometrial cancer (30–70%) (12). Knowledge of the underlying germ line mutation not only allows life-saving intensive-cancer surveillance but also gives asymptomatic family members the opportunity to clarify their carrier status; due to autosomal dominant inheritance offspring of a LS patient has an *a priori* chance of 50% of having inherited the pathogenic MMR mutation.

In contrast to their sporadic MSS CRC LS patients’ tumors typically have a comparatively favorable prognosis and absence of distant organ metastasis; together with the observation that LS cancers are accompanied by an intense immune response with dense lymphocytic infiltrates points to a possible protective effect by the immune system (13).

The revised Bethesda guidelines are probably the most commonly applied criteria to identify individuals with LS, yet many physicians dealing with familial CRC have the impression that LS *per se* remains underdiagnosed (14). An expert group therefore recently suggested to screen all individuals with CRC or endometrial cancer below age 70 by immunohistochemistry or MSI, both of which having similar clinical sensitivity and specificity. In order to discriminate between a hereditary (about 2–3% of all CRC) or a sporadic event (about 15%), tumors with immunohistochemical loss of MLH1 should then be further investigated for MLH1 promoter hypermethylation and targeted BRAF (V600E hotspot mutation) testing to decide on further (germ line) genetic testing. With high-throughput sequencing techniques entering routine genetic testing it is likely that these diagnostic screening algorithms will considerably change in the foreseeable future (15).

## Role of MSI in Sporadic Cancer

**Figure 1:**
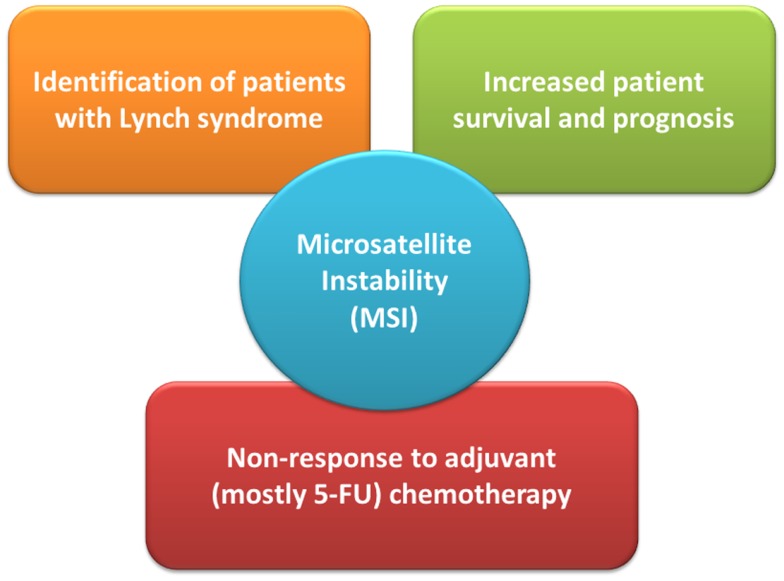
**The role of microsatellite instability (MSI) status in clinical management of colorectal cancer patients**. 5-FU denotes 5-fluoro-uracil.

Since their initial description it became evident that the 15% of sporadic MSI-H CRCs exhibit a distinct clinico-pathological profile, which they largely share with LS-related CRC and which distinguishes them from their MSS counterparts (Figure 1). Already in the seminal work by Thibodeau et al. (2) MSI-H CRC were predominantly located in the proximal colon and associated with increased patient survival and prognosis (2). Most of them were later found to exhibit loss of MLH1 protein expression which could be attributed to epigenetic silencing of the respective promoter, later also referred to as “CpG island methylator phenotype” since it often occurs in the context of global hypermethylation (16, 17). Regarding their molecular-histopathological profile MSI-H CRC display a diploid state, tend to be poorly differentiated, mucinous, and show prominent lymphocytic infiltration (15).

A recent meta-analysis by Guastadisegni et al. who pooled data from 31 studies reporting survival in 12782 patients confirmed the initial observations between MSI-H status and a more favorable prognosis: patients with stages I-IV MSI-H CRC appeared to have a statistically significantly better outcome in terms of overall survival, disease-specific as well as disease-free survival (18). Moreover, results from a recent Norwegian study have shown that MSI status had an independent positive prognostic impact on stage II CRC patients after complete resection (19). How these findings and the inclusion of additional molecular markers may eventually impact on routine clinical and surgical practice, however, remains to be seen.

Guastadisegni et al. also investigated the effect of standard, 5-fluoro-uracil (5-FU) based chemotherapy. In the context of MSI and an underlying (hereditary or sporadic) defect in MMR the use of 5-FU in MSI-H CRC merits particular attention: as demonstrated in numerous *in vitro* studies, inactivation of the MMR system can result in resistance, or rather tolerance (i.e., failure to induce cell-cycle arrest), to 5-FU treatment (4, 20). In line with these observations the meta-analysis found a clear significant beneficial effect of 5-FU therapy in patients with MSS CRC only. Further studies by Des Guetz et al. (meta-analysis) and Sargent et al. provided comparable findings in that relapse-free survival was similar for treated and untreated MSI-H patients demonstrating MSI-H status as a predictive factor of non-response to adjuvant, mostly 5-FU-based chemotherapy (21, 22). The high variability in treatment response observed in the MSI-H CRC group may actually reflect the (in) efficiency of other DNA repair systems, like base-excision repair, to repair/tolerate chemotherapy-induced DNA lesions. Overall, current data advocate CRC patient stratification by determining the tumor’s MMR status, either by testing for MSI or immunohistochemical analysis of MMR proteins, in order to decide on adjuvant chemotherapy on an individual basis.

## Concluding Remarks

As exemplified by the rapidly growing list of cancer genomes analyzed by means of ever more complete as well as cost-effective high-throughput (“next generation”) sequencing methods, a novel era of truly personalized medicine seems to be at hand (23). It is the hope of the author that, despite the inherent difficulties and (data) challenges when trying to get a deeper understanding of biological systems as complex as cancer, the novel “omics” and systems biology approaches will not only allow more comprehensive insights into tumor biology but eventually result in individual patient (tumor)-tailored treatment and, last not least, enable true cancer prevention.

## Conflict of Interest Statement

The author declares that the research was conducted in the absence of any commercial or financial relationships that could be construed as a potential conflict of interest.
